# Broad functional landscape of geminivirus-encoded C4/AC4 protein

**DOI:** 10.1007/s44297-023-00009-6

**Published:** 2023-10-13

**Authors:** Yuzhen Mei, Yaqin Wang, Xueping Zhou

**Affiliations:** 1 State Key Laboratory of Rice Biology and Breeding, Institute of Biotechnology, Zhejiang University, Hangzhou, 310058 Zhejiang China; 2 State Key Laboratory for Biology of Plant Diseases and Insect Pests, Institute of Plant Protection, Chinese Academy of Agricultural Sciences, Beijing, 100193 China

**Keywords:** Geminivirus, C4, AC4, Counter-defense

## Abstract

Geminiviruses induce severe developmental abnormalities in crops and cause severe economic losses worldwide. Owing to the limited coding capacity of the viral genome, geminiviruses produce a limited number of multifunctional effectors to adapt to the host cellular environment. Among these effectors, the geminivirus-encoded C4/AC4 protein is a powerful viral effector with unexpected versatility. Here, we summarize the current knowledge on this remarkable protein and discuss future work on the C4/AC4 protein to provide new insights into plant-geminivirus interactions and integrated control of diseases induced by them.

## Introduction

**Fig. 1:**
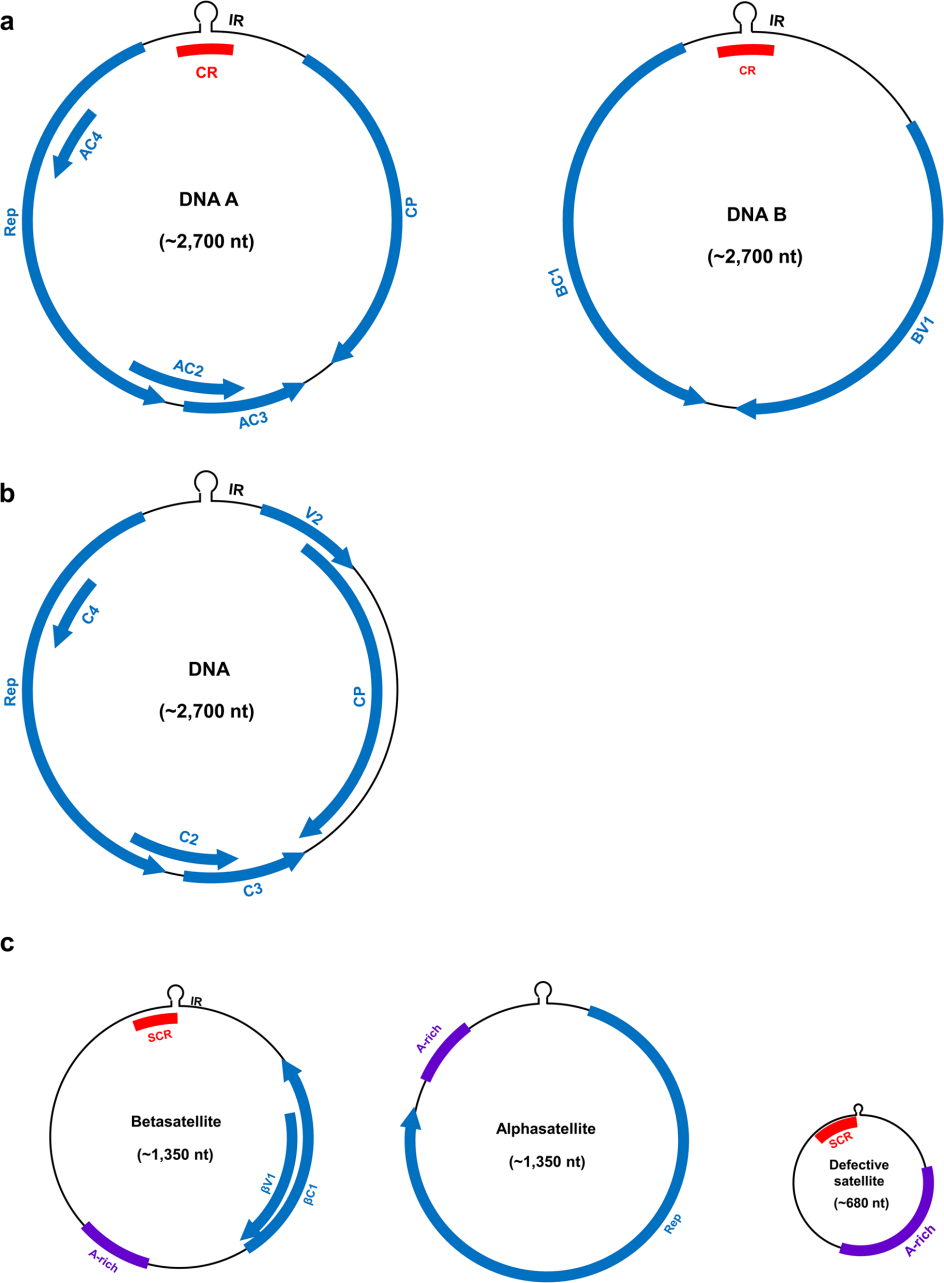
Genomic organization of begomoviruses and their associated satellites. **a** Bipartite begomoviruses encode coat protein (CP), Rep, AC2, AC3, AC4, BV1 and BC1. **b** Monopartite begomoviruses encode CP, V2, Rep, C2, C3 and C4. **c** Betasatellites encoding βC1 and βV1, alphasatellites encoding Rep protein, and defective betasatellite molecules. Abbreviations: A-rich, adenine-rich region; CR, common region; IR, intergenic region; SCR, satellite conserved region

Geminiviruses cause devastating diseases in fields and economic losses in agriculture worldwide [1–8]. The *Geminiviridae* family is divided into 14 genera based on host range, genome structure and insect vectors: *Becurtovirus*, *Begomovirus*, *Capulavirus*, *Citodlavirus*, *Curtovirus*, *Eragrovirus*, *Grablovirus*, *Maldovirus*, *Mastrevirus*, *Mulcrilevirus*, *Opunvirus*, *Topilevirus*, *Topocuvirus*, and *Turncurtovirus* [9]. These viruses encapsidate circular, single-stranded DNA (ssDNA) genomic components that encode 6–8 canonical viral proteins located in both virion and complementary strands [6]. *Begomovirus* is a unique genus with both a monopartite genome and bipartite genome and is also the largest genus in this family [10, 11]. The virion-sense strand DNA encodes the coat protein (CP) and the V2/AV2 protein, and the complementary sense strand DNA encodes the C1/AC1 protein (or replication associated protein, Rep), the C2/AC2 protein (or transcriptional activator protein, TrAP), the C3/AC3 protein (or replication enhancer protein, REn), and the C4/AC4 protein (Fig. 1). Some monopartite begomoviruses associate with satellite molecules, which sometimes provide additional proteins that act as virulence factors [6, 12, 13]. Recent studies have reported that begomoviruses encode several additional small open reading frames (ORFs) [14, 15]. For begomoviruses without satellite association, the C4/AC4 protein functions as a powerful effector with unexpected versatility.

### Geminivirus C4/AC4 functions as a viral symptom determinant

The geminivirus C4/AC4 protein, a small viral effector frequently harboring a consensus N-terminal myristoylation motif, is encoded in an open reading frame (ORF) fully embedded in that encoding the replication initiator protein (Rep/AC1/C1). The geminivirus C4/AC4 protein acts as a symptom determinant to induce viral symptoms [12, 13, 16–21]. For example, transgenic plants constitutively expressing tomato yellow leaf curl virus (TYLCV) *C4* develop virus-disease-like phenotypes [22]. C4 from beet severe curly top virus (BSCTV) induces abnormal cell division through inducing the upregulation of *KRP*, which interacts with and might degrade the cyclin kinase inhibitor (ICK2) to interfere with the mitotic cycle [16]. BSCTV C4 also interacts with CLAVATA 1 (CLV1) to meddle the CLAVATA pathway for cell division regulation [12, 13]. Moreover, C4, encoded by a recombinant virus, tomato leaf curl Yunnan virus (TLCYnV), which has a recombinant origin with tomato yellow leaf curl China virus (TYLCCNV) as the major parent and pepper yellow leaf curl China virus (PepYLCCNV) as the donor of the C4 gene and the intergenic region, is able to interact with SKη kinase and tether nucleus-localized SKη to the plasma membrane to impede cyclin CycD1,1 degradation to induce the transition of the cell from G1 to S phase, which facilitates viral genome replication [17, 18, 23]. Furthermore, a recent study found that divergent symptoms caused by geminivirus-encoded C4 proteins correlate with their ability to bind SKη [24]. This study showed that the C4 proteins from different geminiviruses showed a differential ability to interact with NbSKη, which correlated with their symptom determinant capability [24]. These findings showed that geminivirus C4/AC4 acts as a viral ‘‘oncogene’’, similar to those encoded by animal viruses [25], regulating cell cycle progression and causing developmental defects.

### Geminivirus C4/AC4 represses and manipulates plant defenses

#### C4 suppresses antiviral gene silencing

As a multifunctional effector, geminivirus C4/AC4 proteins have also been identified as gene silencing suppressors. The C4/AC4 proteins encoded by African cassava mosaic virus (ACMV), East African cassava mosaic virus (EACMV) and cotton leaf curl Multan virus (CLCuMuV) have been identified as suppressors of posttranscriptional gene silencing (PTGS) [26–29]. The C4 proteins from TYLCV and tomato leaf curl Guangdong virus (ToLCGdV) and the AC4 encoded by mungbean yellow mosaic virus (MYMV) interact with BARELY ANY MERISTEM 1 (BAM1), which is proposed to promote the intercellular movement of silencing [30–32], to inhibit the spread of silencing. The TLCYnV C4 protein also functions as a transcriptional gene silencing (TGS) suppressor and interacts with DOMAINS REARRANGED METHYLASE 2 (DRM2), a pivotal DNA methyltransferase in the methyl cycle, to interfere with its ability to bind viral DNA to suppress viral genome methylation [33]. Moreover, the C4 protein encoded by CLCuMuV interacts with S-adenosyl methionine synthetase (SAMS), the core enzyme in the methyl cycle, and inhibits its enzymatic activity [28].

### C4/AC4 interferes with different aspects of plant defense

Geminivirus C4/AC4 proteins are able to counter plant defense responses, which are not restricted to antiviral gene silencing, during viral infection. C4 from TYLCV, beet curly top virus (BCTV) and EACMV can shuttle from the plasma membrane to chloroplasts upon artificial activation of PTI to hamper downstream chloroplast-mediated defenses, including salicylic acid (SA) accumulation and SA-dependent responses, which play important roles in host antiviral defenses [34, 35]. The interaction of C4 from TLCYnV with HYPERSENSITIVE INDUCED REACTION 1 (HIR1) inhibits HIR1 dimerization and promotes the degradation of HIR1 to abolish the HIR1-mediated hypersensitive response [36]. Meanwhile, the interaction of TLCYnV C4 with BRI1 KINASE INHIBITOR 1 (BKI1) tethers BKI1 at the plasma membrane and inhibits its dissociation from ERECTA (ER), suppressing ER autophosphorylation and subsequent mitogen-activated protein kinase (MAPK) cascade activation [37]. Recently, CLCuMuV C4 has been shown to inhibit autophagy by binding to the autophagy negative regulator eukaryotic translation initiation factor 4A (eIF4A) to enhance the eIF4A-autophagy-related protein 5 (ATG5) interaction [38].

## Conclusions and outlook

**Fig. 2:**
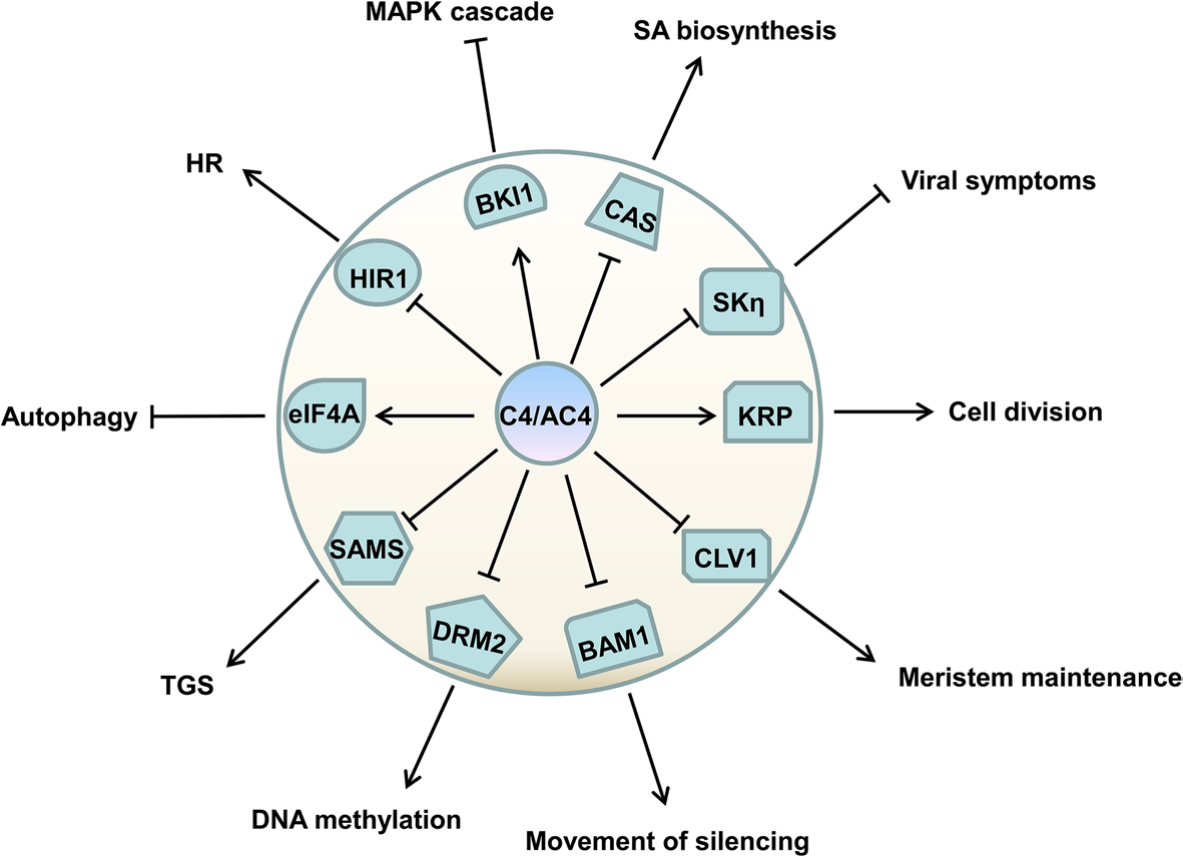
Broad functional landscape of geminivirus-encoded C4/AC4 protein. C4/AC4 proteins from geminiviruses are symptom determinants and interfere with host defense by interacting with host factors

Previous studies uncovered a plethora of functions and different properties of geminivirus C4/AC4 proteins and showed that geminivirus C4/AC4 proteins act as powerful multifunctional effectors that determine viral symptoms or manipulate defense responses (Fig. 2). However, no host factors were found to interfere with C4/AC4 function, and it will be of great significance to unravel the host factors targeting geminivirus C4/AC4 proteins. Further study in this direction would provide additional insight into sustainable field resistance engineering for major crop diseases. Additionally, the annotated or predicted C4/AC4 homologs in other genera (*Becurtovirus*, *Capulavirus*, *Grablovirus*, *Maldovirus*, *Mastrevirus*, *Mulcrilevirus*, *Opunvirus*, *Topilevirus* and *Turncurtovirus*) of *Geminiviridae* are not fully understood. It is worth exploring whether the functions of C4/AC4 proteins from these viruses overlap with those of C4/AC4 proteins encoded by begomoviruses and curtoviruses. Further efforts to research C4/AC4 homologs might provide new insights into intricate plant-geminivirus interactions.

## Data Availability

Data will be made available on request.
